# Unmet need for family planning in Ethiopia and its association with occupational status of women and discussion to her partner: a systematic review and meta-analysis

**DOI:** 10.1186/s40834-020-00121-w

**Published:** 2020-11-20

**Authors:** Solomon Adanew Worku, Yohannes Moges Mittiku, Abate Dargie Wubetu

**Affiliations:** 1 Department of Midwifery, College of Health Science, Debre Berhan University, P.O.Box 445, Debre Berhan, Ethiopia; 2 Department of Nursing, College of Health Science, Debre Berhan University, Debre Berhan, Ethiopia

## Abstract

**Background:**

Unmet need refers to fecund women who either wish to postpone the next birth (spacers) or who wish to stop childbearing (limiters) but are not using a contraceptive method. Many women who are sexually active would prefer to avoid becoming pregnant but are not using any method of contraception. These women are considered to have an unmet need for family planning. Therefore, the objective of this systematic review and meta-analysis is to estimate the pooled prevalence of unmet need for family planning and its association to occupational status of women and discussion to her partner among fecund women in Ethiopia.

**Method:**

A systemic review and meta-analysis was conducted using published and unpublished research on the prevalence of unmet need for family planning and its association to occupational status of women and discussion to her partner among fecund women in Ethiopia. Data extraction was designed in accordance with the Preferred Reporting Items for Systematic Reviews and Meta-Analyses (PRISMA) guidelines. Studies were accessed through electronic web-based search from PubMed, Cochrane Library, Google Scholar, CINAHL, and Embase. All statistical analysis were done using STATA version 14 software using random effects model. The pooled prevalence was presented in forest plots.

**Results:**

A total of 9 studies with 9785 participants were included, and the overall pooled estimated prevalence of unmet need for family planning among fecund women in Ethiopia was 34.90% (95% CI: 24.52, 45.28%). According to subgroup analysis the estimated prevalence of unmet need for family planning in studies conducted in Amhara was 32.98% (95% CI: 21.70, 44.26%), and among married women was 32.84% (95% CI: 16.62, 49.07%). Additionally, housewife women were 1.6 times more likely have unmet need for family planning compared to government employed women (OR: 1.6, 95% CI: 1.29, 1.99). Moreover, women who don’t discuss to partner were 1.87 times more likely to have unmet need for family planning compared to women who had discussion to her partner (OR 1.87; 95% CI: 1.52, 2.31).

**Conclusion:**

The analysis revealed that the overall prevalence of unmet need for family planning among fecund women in Ethiopia was high. Family planning programs should identify strategies to improve communication in family planning among couples and to ensure better cooperation between partners.

## Introduction

Unmet need refers to fecund women who either wish to delay the next birth (spacers) or who wish to stop childbirth (limiters) but are not using a contraceptive method. Many women who are sexually active would desire to avoid becoming pregnant but are not using any method of contraception. These women are measured to have an unmet need for family planning [1–3].

Sub-Saharan Africa, 25% of women of reproductive age who are married or in a union have an unmet need for family planning. Also, four countries in Latin America and the Caribbean, eight countries in Asia and four countries in Oceania have an unmet need for family planning above 20% [4, 5].

Ethiopia has among the highest levels of unmet need for contraception in Africa. The 2011 Ethiopia Demographic and Health survey (EDHS) found that 25.3% of women had unmet need for FP, 16.3% for spacing and 9% for limiting. Unmet need for both spacing and limiting is greater among rural residents than their urban counter parts. The over-all unmet need for family planning among urban and rural residents is 15 and 27.5% respectively [6]. Report from EDHS 2016 discloses that 58% of now married women age 15–49 have a request for family planning. Thirty-six percent of currently married women are already using a contraceptive method either to space (22%) or to limit births (14%). Unmet need for currently married women age 15–49 is lowermost in Addis Ababa (11%) and maximum in Oromia region (29%) [7].

In Ethiopia, different studies have been conducted to determine the prevalence of unmet need for family planning and associated factors. The findings of these disjointed studies familiar that there was a great inconsistency in the prevalence of unmet need for family planning across the regions of the country. Concerning associated factors, these studies revealed that different maternal and health service related factors influenced unmet need for family planning; place of residence [8, 9], educational status of women [9–12], occupational status of women [8, 10, 12, 13], partner educational status [9, 10], having a discussion to health provider [8, 10, 14], and having a discussion to her partner [10, 12, 13], were some of the factors related with unmet need for family planning. After these factors, we selected the two factors (occupational status of women and having a discussion to her partner) to see their consequence on unmet need for family planning.

Reducing the proportion of unmet need for family planning has major role in preventing maternal and child health problems. To reduce the proportion of unmet need for family planning, knowing the current level and its determinants is a prerequisite. This systematic review and meta-analysis was conducted to estimate the pooled prevalence of unmet need for family planning and its association to occupational status of women and discussion to her partner among fecund women in Ethiopia. This study can also be useful for other researchers who are interested to conduct further studies. It can also be valuable to the organization working in family planning sector to know the factors influencing unmet needs and conduct necessary programs.

## Methods

### Study design and search strategy

A systemic review and meta-analysis was conducted using published and unpublished research on the prevalence of unmet need for family planning and its association to occupational status of women and discussion to her partner among fecund women in Ethiopia. Cochrane library, PubMed, EMBASE, HINARI, and Google Scholar was systematically searched using the following terms/phrases: “prevalence of unmet need for family planning in Ethiopia”, “unmet need for family planning OR Ethiopia”, “unmet need for family planning AND Ethiopia”. All published and unpublished articles up to March 2019 were included in the systematic review. Additionally, we observed the reference lists of published studies to identify additional articles. Our literature search strategy, selection of publications, data extraction, and the reporting of results for the review were designed in accordance with the Preferred Reporting Items for Systematic Reviews and Meta-Analyses (PRISMA) guidelines [15].

### Study selection and eligibility criteria

We included all studies that were conducted on the prevalence of unmet need for family planning among fecund women in Ethiopia. The participants were fecund women whose age is 15–49 years. We included all study types that were published in the form of journal articles, master’s thesis, and dissertations in English.

### Quality assessment and critical appraisal

Qualities of each article were assessed by using a critical appraisal tool for use in systematic reviews for prevalence study [16]. The methodological quality and eligibility of the identified articles were also assessed by three reviewers (SA, YM, and AD) and disagreements among reviewers were fixed accordingly with discussion. Data were extracted using pre piloted data extraction form which was developed by the authors. SA and YM conducted the primary data extraction and then SA, and AD examined the extracted data independently. Any disagreement and inconsistencies were resolved by discussion and consensus.

### Data analysis and synthesis

The extracted data were entered into computer through command window of STATA v.14 and the analysis was performed using STATA v.14. A random effects model was used to estimate the overall pooled prevalence. An important statistical issue in meta-analysis is handling of heterogeneity among studies. DerSimonian and Lairdmethod, which assumes heterogeneity across studies, is the most common method for using random effects model in meta-analysis [17, 18]. A random effects meta-analysis is also recommended for use when heterogeneity between studies exists. The heterogeneity of studies was checked using *I*^2^ test statistics. *I*^2^ statistics is used to quantify the percentage of total variation in study estimate due to heterogeneity. *I*^2^ statistics ranges from 0 to 100%. A value of 0% indicates no observed heterogeneity while 100% indicates significant heterogeneity. A *p* value less than 0.05 was used to declare heterogeneity. In this meta-analysis, *I*^2^ values were found to be high (> 75%). Since this value is definite indicative of significant high heterogeneity, we conducted the analysis with a random effects model with 95% CIs as opposed to the fixed effect model to adjust for the observed variability. Moreover, presence of heterogeneity was also assessed by subgroup analysis and Meta regression.

Visual assessment of publication bias was conducted using funnel plot. Asymmetry of the funnel plot is an indicator of publication bias. Egger’s and Begg’s tests were also conducted to check potential publication bias. A 푝 value less than 0.05 was used to declare statistical significance of publication bias. Additionally, sensitivity analysis was also done to assess whether the pooled prevalence estimates were influenced by individual studies.

## Results

### Selection and identification of studies

**Fig. 1:**
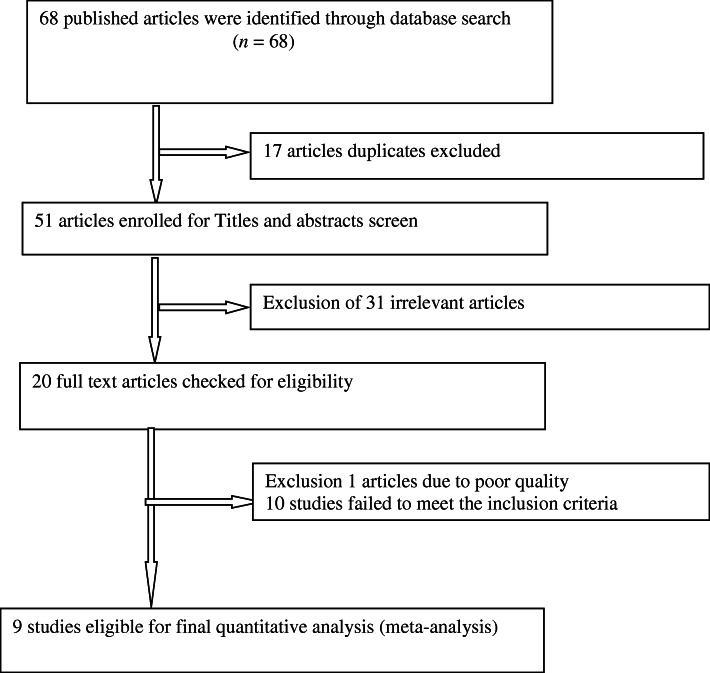
Flowchart of study selection for meta-analysis of prevalence of unmet need for family planning among fecund women in Ethiopia, 2019

A total of 68 studies were identified from the literature search. Of these studies, 17 articles duplicate records were identified and removed. Reviewing of titles and abstracts resulted in exclusion of 31 irrelevant articles. After assessing the full texts of the remaining articles, additional 1 articles were excluded due to poor quality. Moreover, based on the inclusion and exclusion criteria for entry into the study a total of 10 studies were excluded as they did not meet the inclusion criteria. Then, a total of nine unique studies were eligible and enrolled for final analysis (Fig. 1).

### Characteristics of included studies

A total of 9 studies with 9785 participants included in this meta-analysis are summarized in Table 1. The studies were conducted from 2011 to 2019 in different region of the country. Among 9 studies five of them [8, 10, 11, 13, 19] were conducted in Amhara, two study [14, 20] were in Tigray, and the other 2 studies [9, 12] were in other region of the country. All studies were cross-sectional study conducted among married women, reproductive age women, and extended postpartum women in Ethiopia. The study with minimum and maximum sample size was conducted in Oromia and Southern Nations, Nationalities, and peoples’ region, respectively [9, 12]. In addition, out of all studies enrolled in this meta-analysis five studies [8–10, 14, 19] were conducted among married women, three studies were conducted among reproductive age group women, while the remaining study [11–13, 20] were conducted among extended postpartum women (Table 1).

**Table 1:** Characteristics of studies included in meta-analysis of unmet need for family planning in Ethiopia and its association with occupational status of women and discussion to her partner, 2019

No	Author/s (Reference)	Year of publication	Study design	Study area	Sample size	Included population	Study population	Response rate	Prevalence %
1	Genet et al. [8]	2015	Cross sectional	Ethiopia	551	506	Married	99.1%	17.4
2	Gebre et al. [14]	2016	Cross sectional	Ethiopia	510	510	Married	100%	21.4
3	Molla and Belete [19]	2011	Cross sectional	Ethiopia	692	692	Married	100%	47.3
4	Mekonnen and Worku [9]	2011	Cross sectional	Ethiopia	5746	5746	Married	100%	52.4
5	Dejenu et al. [10]	2013	Cross sectional	Ethiopia	770	756	Married	98.1%	25.6
6	Tegegn et al. [11]	2017	Cross sectional	Ethiopia	383	382	Extended postpartum women	99.7%	44
7	Gebrecherkos et al. [20]	2018	Cross sectional	Ethiopia	400	400	Reproductive age women	100%	41.8
8	Mota et al. [12]	2015	Cross sectional	Ethiopia	382	382	Reproductive age women	100%	33.3
9	Worku et al. [13]	2019	Cross sectional	Ethiopia	411	411	Reproductive age women	100%	30.9

### Prevalence of unmet need for family planning

**Fig. 2:**
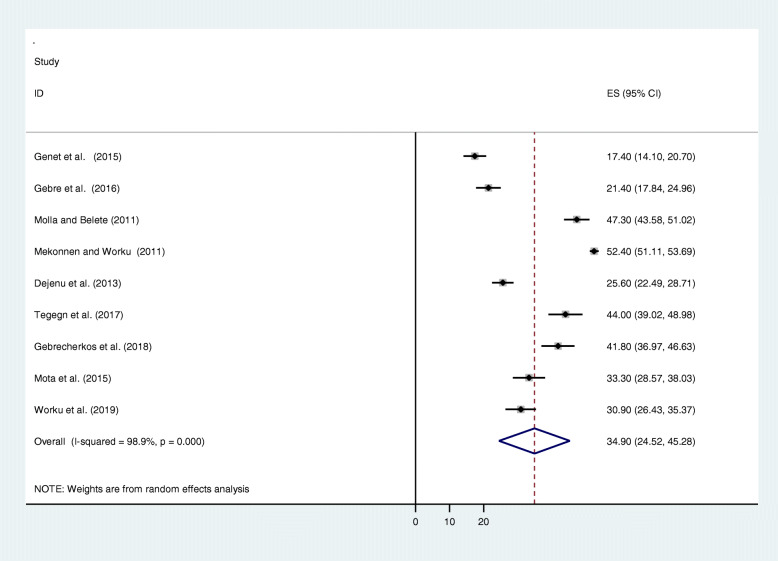
Forest plot showing the pooled prevalence of unmet need for contraceptive among fecund women in Ethiopia, 2019

The pooled prevalence using the fixed effect model showed significant heterogeneity between the studies. Hence, we performed the analyses using random effects model. Using random effects model, the estimated pooled prevalence of unmet need for family planning among fecund women reported by the 9 studies was 34.90% (95% CI: 24.52, 45.28%) with significant heterogeneity between studies (*I*^2^ = 98.9%, *p* = 0.000). The pooled prevalence of unmet need for family planning presented using forest plot (Fig. 2).

**Fig. 3:**
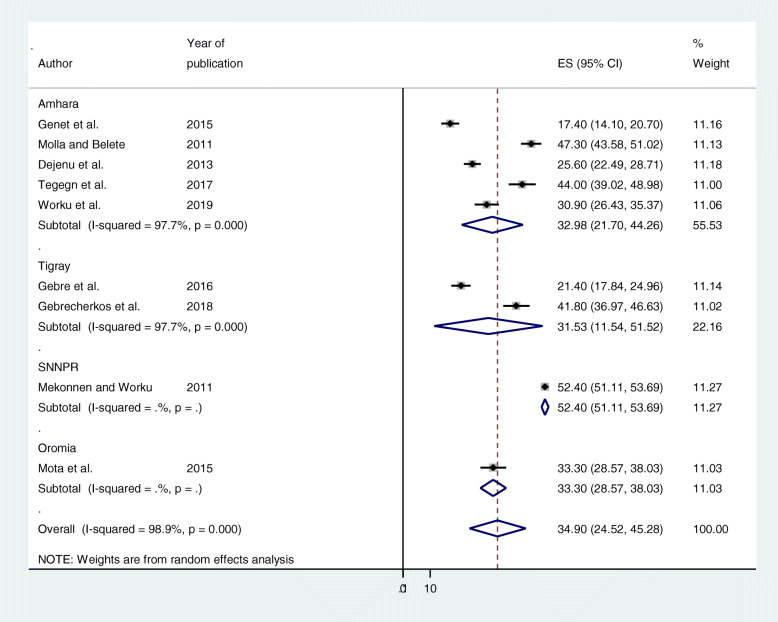
Subgroup analysis by region on the prevalence of unmet need for family planning among fecund women in Ethiopia, 2019

**Fig. 4:**
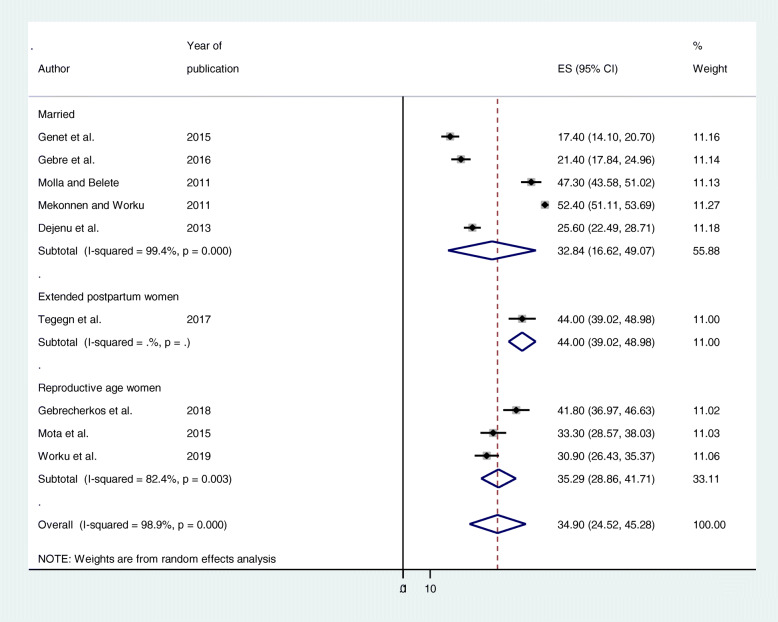
Subgroup analysis by study population on the prevalence of unmet need for family planning among fecund women in Ethiopia, 2019

Subgroup analysis by study area, and study population was conducted to assess the potential heterogeneity between studies. Of the 9 studies, the estimated unmet need for family planning prevalence found in studies conducted in Amhara (32.98% (95% CI: 21.70, 44.26%), *I*^2^ = 97.7%, *p* = 0.000), and studies conducted in Tigray, was 31.53% ((95% CI: 11.54, 51.52%), *I*^2^ = 97.7%, *p* = 0.000) (Fig. 3). In terms of study population, the estimated unmet need for family planning prevalence in studies conducted among married women (32.84% (95% CI: 16.62, 49.07%) *I*^*2*^ = 99.4%, *p* = 0.000), and studies conducted among reproductive age group women was 35.29% ((95% CI: 28.86, 41.71%), *I*^*2*^ = 82.4%, *p* = 0.003) (Fig. 4).

In addition, to identify the possible sources of heterogeneity univariate meta-regression was conducted by considering the sample size and year of publication as covariates. The result showed that none of them were found to be statistically significant (Table 2).

**Table 2:** Meta-regression analysis of factors with heterogeneity of the prevalence of unmet need for family planning among fecund women in Ethiopia, 2019

Heterogeneity source	Coefficients	Std. err.	푝 value
Sample size	0.0035064	0.002933	0.277
Publication year	−0.1702263	1.804313	0.928

### Publication Bias

**Fig. 5:**
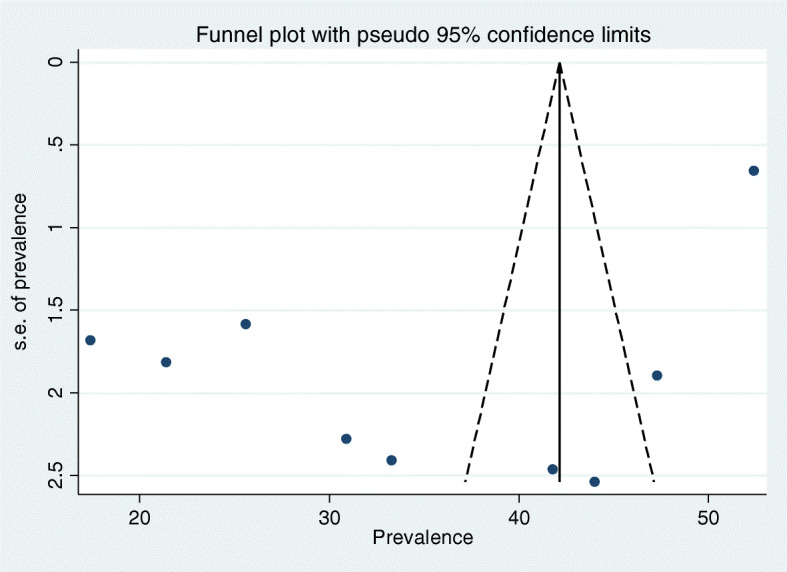
Funnel plots to test the publication bias of the 9 studies, 2019

Presence of publication bias was examined using funnel plots and tests (Egger’s and begs). In this meta-analysis funnel plots and tests indicated evidence of publication bias. Each point in funnel plots represents a separate study and asymmetrical distribution is evidence of the existence of publication bias [21]. First, each study’s effect size was plotted against the standard error and visual inspection of the funnel plot suggests some asymmetry, as six studies lay on the left side and three studies on the right side of the line representing the pooled prevalence (Fig. 5). We also performed Egger’s and Begg’s tests to investigate publication bias. The result of these tests showed significant evidence of publication bias (*p* value < 0.05).

### Sensitivity analysis

**Fig. 6:**
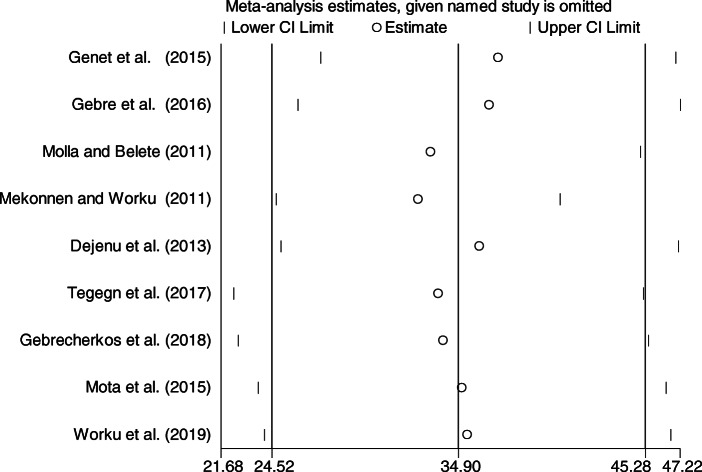
Result of Sensitivity analysis of the 9 studies, 2019

The result of sensitivity analysis using random effects model suggested that no single study unduly influenced the overall prevalence estimate of unmet need for family planning among fecund women (Fig. 6).

### Association between occupational status of women and unmet need for family planning

In this meta-analysis, we examined the association between women occupation and unmet need for family planning by using four available studies [8, 10, 12, 13]. The findings from these four studies revealed that the unmet need for family planning was significantly associated with women occupation. Accordingly, the likelihood of unmet need for family planning was 1.6 times higher among house wife as compared to women’s who have government employed (OR: 1.6, 95% CI: 1.29, 1.99). High heterogeneity (I^2^ = 76.7% and *p* value < 0.005) was observed across the included studies; hence, a random effect meta-analysis model was used to examine the association between women’s occupation and unmet need for family planning. For this analysis, we also assessed publication bias using Begg’s and Egger’s tests, the result of the test statistics indicated that there was no possible presence of statistically significant publication bias (*p* = 0.174 and (*p* = 0.132) respectively.

### Association between women discussion with her partner and unmet need for family planning

Three studies, which examined the association between women discussion with her partner and unmet need for family planning were considered to determine the association between unmet need for family planning and women discussion with her partner (10, 12 13). In this study, the pooled odds ratio indicated that women discussion with her partner was positively associated with unmet need for family planning (OR: 1.87, 95% CI: 1.52, 2.31). In this meta-analysis, high heterogeneity (*I*^2^ = 64.9% and *p* value < 0.036) was observed across the studies hence, a random effect meta-analysis model was employed to estimate the pooled effect. We also assessed publication bias using Begg’s and Egger’s tests, the result of the test statistics indicated that there was no possible presence of statistically significant publication bias (*p* = 0.497 and (*p* = 0.433) respectively.

## Discussion

We conducted this systematic review and meta-analysis to estimate the pooled prevalence of unmet need for family planning in Ethiopia and its association with occupational status of women and discussion to her partner. The pooled prevalence of unmet need for family planning in Ethiopia was 34.90% (95% CI: 24.52, 45.28%). The overall prevalence indicated in this meta-analysis is similar the study conducted in Burundi (32.4%), and Nagpur city in India (31.6%) [21, 22]. In addition, this finding is higher than the study conducted in Botswana (9.6%), rural Burkina Faso (18.26%), Urban Cameroon (20.4%), Zambia (25.5%), Nnewi, south-east Nigeria (21.4%), and Bangladesh (22.4%) [22–27]. On the other hand, our finding is lower than the study conducted in Eastern Sudan (44.8%.), Angola (51.7%), North West Region, Cameroon (46.6%), Kenya (46%), Guatemala (67.6%), India Belgaum (64%), Pakistan (96.6%), and Nepal during the first 2 years postpartum (52%) [22, 28–32]. The possible explanations for the above variations could be due to methodological differences (sampling of study participants), and health service utilization.

We also performed subgroup analysis by study area, and study population was conducted to assess the potential heterogeneity between studies. The estimated unmet need for family planning prevalence found in studies conducted in Amhara (32.98%), and studies conducted in Tigray, was 31.53%. In terms of study population, the estimated unmet need for family planning prevalence in studies conducted among married women (32.84%), and studies conducted among reproductive age group women was 35.29%.

The current meta-analysis was also examined the association between women discussion with her partner and unmet need for family planning, and association between occupational status of women and unmet need for family planning in Ethiopia. Accordingly, women discussion with her partner was positively associated with unmet need for family planning, and occupational status of women was positively associated with unmet need for family planning. Women who had not a discussion with her partner were almost 1.87 times more likely to have unmet need for family planning as compared to women who had a discussion to her partner. This finding is consistent with the studies conducted in Botswana [23], Urban Cameroon [25], and North West Region, Cameroon [30]. This could be due to the fact that couples where both partners reported communicating with each other regarding desired number of children and family planning use were more likely to use contraception compared to couples that did not communicate. Spousal communication regarding family planning would be an effective way to motivate partner for supporting and using contraceptives [33]. Unmet need for family planning is higher among housewife women than among government employed women. Women who are housewife were almost 1.6 times more likely to have unmet need for family planning as compared to government employed women. This finding is in agreement with studies conducted in Eastern Sudan [28]. The reason for this may be due to economic and educational concern. Housewives do not have their own monthly income and they are dependent on their partner. Due to this, they may believe that they can’t afford expenses to use contraceptive methods. A woman’s educational attainment has the greatest impact on her contraceptive behavior.

## Conclusion and recommendation

The overall prevalence of unmet need for family planning among fecund women in Ethiopia was high. Women occupational status and have a discussion with her partner were significantly associated with unmet need for family planning. Family planning programs should identify strategies to improve communication in family planning among couples and to ensure better cooperation between partners.

## Data Availability

Data will be available upon request of the corresponding author.
